# Neurotensin Receptor 1 Regulates HER4 Tyrosine Phosphorylation in Lung Cancer Cells

**DOI:** 10.3390/biology15090686

**Published:** 2026-04-28

**Authors:** Terry W. Moody, Irene Ramos-Alvarez, Robert T. Jensen

**Affiliations:** 1 Center for Cancer Training, National Cancer Institute, Bethesda, MD 20892, USA; 2 Digestive Disease Branch, National Institute of Diabetes and Digestive Disease, Bethesda, MD 20892, USA; irene.ramosalvarez@nih.gov (I.R.-A.); robertj@bdg10.niddk.nih.gov (R.T.J.)

**Keywords:** neurotensin, NTSR1, HER4, transactivation, ERK phosphorylation, lung cancer proliferation, SR48692, ibrutinib, NRG1

## Abstract

Neurotensin (NTS) binds to the G protein-coupled receptors (GPCRs) NTSR1 and NTSR2. NTSR1 regulates transactivation of the EGFR, HER2, and HER3, but its effects on HER4 are unknown. By Western blot, NTSR1 and HER4 were present in six lung cancer cell lines examined. In NCI-H522 or NCI-H661 cells, adding NTS increased phosphorylation (P) of tyrosine (Y) 1284 on HER4. Because SR48692 antagonized NTS’s ability to increase P-HER4 or P-ERK, NTSR1 may play an important role in NSCLC. SR48692, HER4 siRNA, reactive oxygen species inhibitors, and the tyrosine kinase inhibitor ibrutinib inhibited NTS-induced P-HER4. Adding NTS to NCI-H661 cells increased the formation of HER4/HER4, HER4/ EGFR, and HER4/HER2 dimers. Adding NTS to NSCLC cells increased both P-ERK and P-AKT, which were inhibited by PD98059 and LY294002, respectively. The growth of NCI-H522 or NCI-H661 cells was stimulated by NTS or neuregulin 1 (NRG1), a HER4 ligand, but inhibited by SR48692 or ibrutinib. The results indicate that NTSR1 regulates HER4 transactivation, thereby increasing the proliferation of lung cancer cells.

## Simple Summary

The G-protein-coupled receptor (GPCR) for neurotensin (NTS) phosphorylates receptor tyrosine kinases (RTK) for the EGFR, HER2, and HER3. While the EGFR, HER2, and HER3 function as oncogenes in lung cancer, the role of HER4 has not been assessed. Because HER4 is metabolized, it inhibits the growth of breast, bladder, liver, and prostate cancers. Adding NTS to lung cancer cells increases tyrosine phosphorylation (P) of HER4 and ERK, thereby stimulating cell growth of lung cancer. The effects of NTS were antagonized by SR48692 (NTSR1 antagonist), HER4 siRNA, ibrutinib (tyrosine kinase inhibitor), reactive oxygen species (ROS) inhibitors, and GM6001 (MMP inhibitor). HER4 forms heterodimers with the EGFR and HER2. The results indicate that NTS and NRG1 stimulate HER4 phosphorylation in lung cancer, thereby increasing proliferation. Phosphorylations of HER4 are inhibited by SR48692 and ibrutinib, which reduce proliferation.

## 1. Introduction

Neurotensin (NTS) is a 13-amino acid peptide [1] which binds to G-protein-coupled receptors (GPCR). NTSR1 binds NTS with high affinity, whereas NTSR2 binds NTS with low affinity [2,3]. In contrast, NTSR3 is not a GPRC but is sortolin [4]. The NTS/NTSR1 complex interacts with Gq11, resulting in the activation of phospholipase (PL)C, which metabolizes phosphatidyl (PI) 4,5 bisphosphate (PIP2) to diacylglycerol (DAG), resulting in activation of protein kinase C (PKC) and inositol trisphosphate (IP3), which elevates cytosolic calcium (Ca^2+^) [5]. When NTS binds to NTSR1, Rho GTPases, NFκB, FAK, and Wnt signaling pathways are activated. E-cadherin is increased after NTS is added to liver cancer cells, leading to epithelial-to-mesenchymal transition [6]. The actions of NTS are antagonized by the small molecule SR48692 [7]. NTSR1 has 418 amino acids and 7 transmembrane (TM) domains. NTS as well as NTS^8–13^ bind to the top of a binding pocket and interact with NTSR1 extracellular loops 2 and 3, as well as transmembrane (TM) loops 6 and 7 [8]. SR48692 binds deep into the NTSR1 pocket and antagonizes the effects of NTS. BMS-553 is a biased agonist for NTSR1, which impairs Gq11 signaling [9].

Lung cancer subtypes include small cell lung cancer (SCLC), which kills approximately 15,000 U.S. citizens annually, and non-SCLC (NSCLC), an epithelial tumor, which kills approximately 115,000 U.S. citizens annually [10]. SCLC cell lines contain high levels of NTS [11,12]. NTS and NTS^8–13^ but not NTS^1–8^ or levocabastine (Levo) bind with high affinity to both SCLC and NSCLC cells [13]. Adding NTS to cancer cells increases the phosphorylation (P) of extracellular signal-related kinases 1 and 2 (ERK) [14]. P-ERK increases the expression of c-fos and c-jun [15], altering gene expression. NTS stimulates, whereas SR48692 inhibits the growth of lung cancer cells in vitro and in vivo [16]. High NTSR1 expression is associated with decreased survival in lung cancer patients [17].

NSCLC cells but not SCLC cells express receptor tyrosine kinases (RTKs) such as EGFR, HER2, HER3, and HER4 [18]. When the EGFR binds ligands such as EGF or TGFα, PI3K or phospholipase C become phosphorylated [19]. Activated EGFRs phosphorylate themselves at Y1068, leading to ERK activation and cancer proliferation. NSCLC patients with L858R EGFR mutations respond to tyrosine kinase inhibitors (TKI), such as erlotinib or gefitinib [20]. HER4 is activated by neuregulin1 (NRG1), increasing P-HER4 [21]. The activation of HER4 is impaired by the TKI ibrutinib [22]. After activation, HER4 can dimerize with itself or other RTKs, leading to increased lung cancer proliferation [21].

NTSR1 regulates the EGFR transactivation in colon, gastric, liver, lung, neuroendocrine, and prostate cancer cells, increasing PY1068-EGFR [5,23,24,25,26,27]. Adding NTS to NSCLC cells increases PY1248-HER2 and PY1289-HER3, respectively [28,29,30]. While EGFR, HER2, and HER3 are oncogenes in lung cancer, HER4 is metabolized. The HER4 metabolites inhibit the growth of breast [31], bladder, liver, and prostate cancers. In contrast, adding NTS to cancer cells increases the growth of brain, colon, gastric, head and neck, lung, ovary, and thyroid cancers [32,33]. In this communication, the ability of NTS to increase HER4 tyrosine phosphorylation and proliferation was investigated using NSCLC cells.

## 2. Materials and Methods

### 2.1. Materials

NTS, NTS^1–8^, NTS^8–13^, and SR48692 were purchased from Bio-Techne Corp (Minneapolis, MN, USA). NCI-H358, NCI-H522, A549, NCI-H661, NCI-H1299, and NCI-H2073 cell lines were purchased from American Type Culture Collection (Rockville, MD, USA). Anti-HER4, anti-EGFR, anti-HER2, anti-HER3, anti-PY^1284^HER4, anti-PT202, PY204-ERK, anti-PS473AKT, and anti-tubulin antibodies were from Cell Signaling Technology, Inc. (Beverly, MA, USA). RPMI-1640, FBS, Trypsin/EDTA, 4–20% Tris–Glycine gels, and DMSO were from Invitrogen (Carlsbad, CA, USA). Inhibitors such as PP2, GM6001, ibrutinib, DPI, Tiron, or N-acetylcysteine were purchased from Sigma–Aldrich (St. Louis, MO, USA). West Dura extended substrate, Super Signal Femto enhanced chemiluminescent detection reagent, and Tiron were from Thermo-Fisher Scientific (Rockford, IL, USA). Stabilized goat anti-rabbit IgG peroxidase conjugate was from Pierce Biotechnology, Inc. (Rockford, IL, USA). Nitrocellulose membranes were purchased from Schleicher and Schuell Bioscience, Inc. (Keene, NH, USA). Laemli buffer was purchased from Sigma-Aldrich, St. Louis, MO, USA. Immunoprecipitations were performed using protein A/G agarose (Santa Cruz Biotechnologies, Paso Robles, CA, USA). BCA protein reagent was purchased from Thermo Fisher (Waltham, MA, USA). HER4 siRNA and NRG1 siRNA were purchased from Dharmacon (Lafayette, CO, USA).

### 2.2. Cell Culture

NSCLC cell lines NCI-H322, NCI-H522, A549, NCI-H661, NCI-H1299, and NCI-H2073 were cultured in DMEM containing 10% FBS and 1% penicillin/streptomycin at 37 °C in 5% CO_2_/95% air. The cells were used in the exponential growth phase and were mycoplasma-free. They were split twice weekly, 1/10 with trypsin/EDTA.

### 2.3. Western Blotting and Immunoprecipitation

NCI-H522 and NCI-H661 cells were cultured in 6-well plates or 10 cm dishes. For siRNA experiments, 10 nM HER4 siRNA or 10 nM NRG1 siRNA was added to NCI-H522 or NCI-H661 cells in 6-well plates for 24 h. The cells were placed in 5 mL of RPMI-1640 containing 5 μg/mL bovine insulin, 10 μg/mL apo-transferrin, and 50 nM Na_2_SeO_3_ (SIT) medium, and inhibitors such as 0.5 μM SR48692, 10 μM PP2, 10 μM GM6001, 0.01 μM ibrutinib (I), 10 μM diphenylene iodonium (DPI), 5 mM Tiron (Tir), 10 μM PD59059, 10 μM LY294002, 10 mM N-acetylcysteine (NAc) were added for 30 min at 37 °C. NTS (0.1 μM) was added to the NSCLC cells for 10 min at 37 °C. The 6-well plates were placed on ice and rinsed with 1 mL of TBS. The cells were then placed in 0.2 mL of lysis buffer containing 50 mM Tris-HCl (pH 7.4), 150 mM NaCl, 1% NP-40, 1% sodium deoxycholate, 1 mM PMSF, 0.2 mM sodium vanadate, and 0.5 mM EGTA. The extracts were sonicated and centrifuged at 10,000× *g* for 1 min at 4 °C. The supernatant protein was determined using the BCA protein reagent. For Western blots, 40 μg of protein was loaded onto 4–20% SDS gels. The protein on the gels was transferred to nitrocellulose for 16 h at 5 °C. The nitrocellulose was treated with a washing buffer containing 0.05% Tween-20 and 5% milk 3 times, followed by the addition of anti-P-HER4, anti-HER4, anti-P-ERK, anti-NTSR1, anti-P-AKT, or anti-tubulin antibodies (1:2000) for 16 h at 4 °C. The blots were treated with washing buffer 3 times, followed by secondary Ab (1:000) for 1 h at 25 °C. The blots were developed with SuperSignal West Femto chemiluminescent substrate. Protein band densities were determined using Gene Tools software (Syngene, Frederick, MD, USA).

For immunoprecipitation experiments, 600 μg of protein was incubated with 4 μL of anti-EGFR, anti-HER2, anti-HER3, or anti-HER4 antibody for 30 min, followed by overnight incubation with 20 μL of protein A/G agarose. Samples were washed 3 times, resuspended in 12 μL of 2× SDS Laemmli buffer, and boiled before electrophoresis. Western blots were processed as described above and assayed using anti-P-HER4.

### 2.4. Cytosolic Ca^2+^

NCI-H662 cells were treated with trypsin/EDTA and centrifuged. The pellet was resuspended in 10 mL of R10 and incubated with 5 μM Fura2AM at 37 °C for 30 min. The cells were centrifuged at 1000× *g*, and the pellet was resuspended in 10 mL SIT. The cell suspension (2 mL) was placed in a glass cuvette, and NTS analogs were added. The fluorescence excitation was at 340 and 380 nm, and the emission was determined at 510 nm for 4 min using a Perkin Elmer L2 spectrofluorometer. Each experiment was repeated 4 times.

### 2.5. Proliferation Assay

The proliferation of NSCLC cells was investigated using the clonogenic and 3-(4,5-dimethyl,-diphenyl-2H-tetrazolium bromide (MTT) assays. The clonogenic base layer contained 3 mL of 5% FBS in SIT medium with 0.5% agarose in 6-well plates in the clonogenic assay. The upper layer contained 3 mL of SIT medium with 0.3% agarose, 10^5^ NCI-H661 or NCI-H522 cells, and NTS (0.1 μM) with or without SR48692 (1 μM) or ibrutinib (0.01 μM). One ml of p-iodonitrotetrazolium violet was added after 2 weeks. The next day, the number of colonies larger than 50 μm in diameter was counted using an Omnicon image analysis system. For the MTT assay, NCI-H522 or NCI-H661 cells (105) were placed in 96-well plates with 100 μL of SIT medium in the presence or absence of SR48692 or ibrutinib. After 24–48 h, 15 μL of MTT (1 mg/mL) was added, and 150 μL of DMSO was added after 4 h. The absorbance was determined at 570 nm.

### 2.6. Statistics

The data were analyzed using Prism 10’s descriptive statistics, and the mean value + S.D. from at least 3 determinations was indicated. Statistical significance *p* < 0.01, **; *p* < 0.05, * was calculated using ANOVA (the posttest was Dunnett’s multiple comparison). Western blot band densities with drugs were divided by control bands with no additions to calculate the % stimulation.

## 3. Results

### 3.1. Cell Line Proteins Were Evaluated by Western Blot

**Figure 1:**
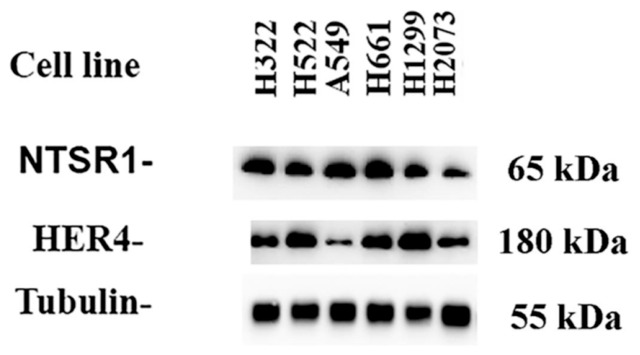
Western blot of NSCLC cell lines. NTSR1, HER4, and tubulin were present in all cell lines examined. This experiment is representative of 2 others.

Figure 1 shows that high concentrations of NTSR1 were present in 5 NSCLC cell lines examined, but moderate concentrations in NCI-H2073. High levels of HER4 were present in NCI-H322, NCI-H522, NCI-H661, and NCI-H1299, whereas moderate levels were present in A549 and NCI-H2073 cells. High levels of the tubulin control were present in all cell lines examined. See original images in Supplemental Figure S1. Because NCI-H522 and H661 cells had high densities of HER4 and NTSR1, they were used in subsequent studies.

### 3.2. Cytosolic Ca^2+^

**Figure 2:**
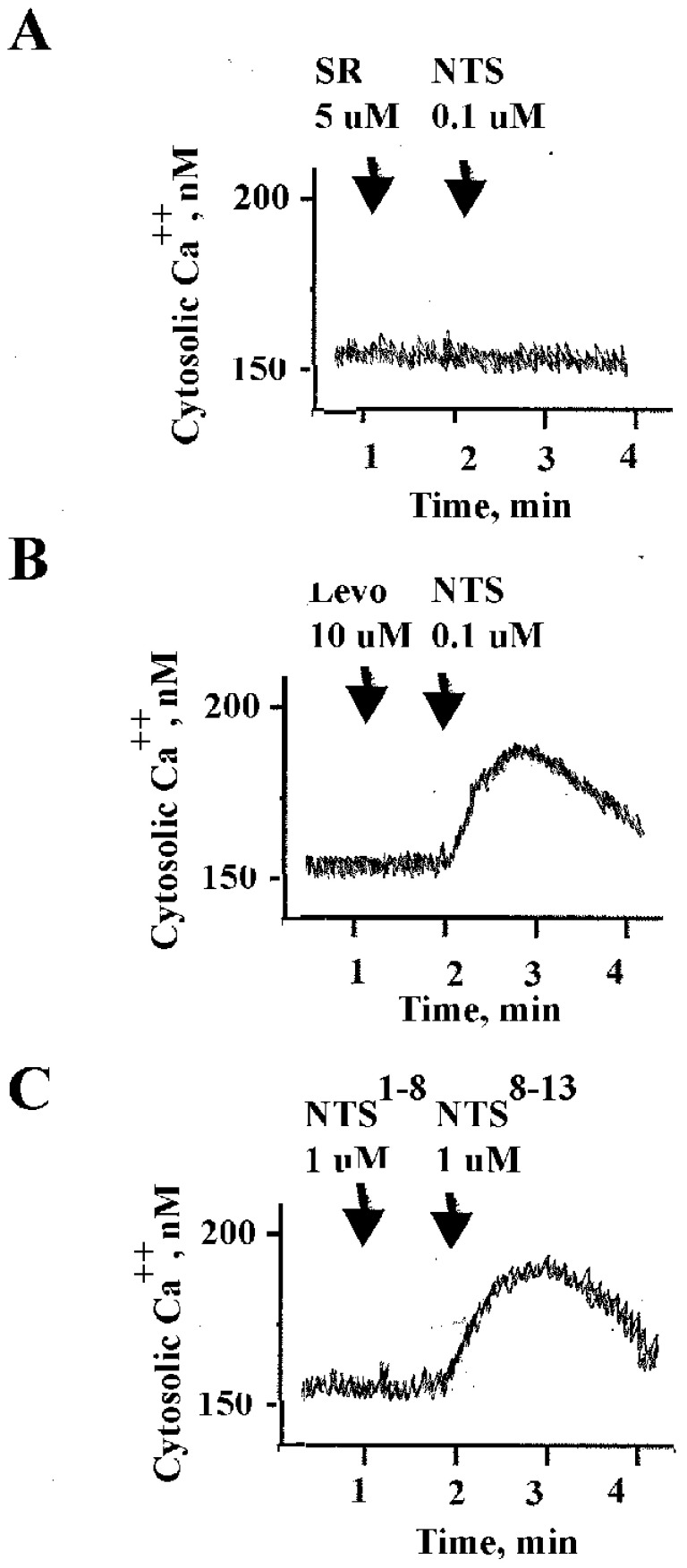
Cytosolic Ca^2+^. (**A**) The ability of SR48692 to block the increase in Ca^2+^ by NTS was investigated. (**B**) The ability of Levo or NTS to alter cytosolic Ca^2+^ in Fura-2 AM-loaded NCI-H661 cells was investigated. (**C**) The ability of NTS^1–8^ or NTS^8–13^ to alter cytosolic Ca^2+^ was examined in NCI-H661 cells. This experiment is representative of 2 others.

The effects of NTS analogs on cytosolic Ca^2+^ were investigated. Using Fura-2 loaded NCI-H661 cells, SR48692 had no effect on cytosolic Ca^2+^ but inhibited the effects of 0.1 μM NTS (Figure 2A). Levocabastine (Levo) had no effect on cytosolic Ca^2+^, but adding 0.1 μM NTS increased the cytosolic calcium rapidly for the first 15 s, but was followed by a slow decline. (Figure 2B). NTS^1–8^ had little effect on cytosolic Ca^2+^; however, NTS^8–13^ (1 μM) increased the cytosolic Ca^2+^ (Figure 2C) strongly. The results indicate that NTS and NTS^8–13^ are NTSR1 agonists, whereas NTS^1–8^ or Levo are inactive, and SR48692 is an antagonist.

### 3.3. NTS Ligands and HER4 Phosphorylation

**Figure 3:**
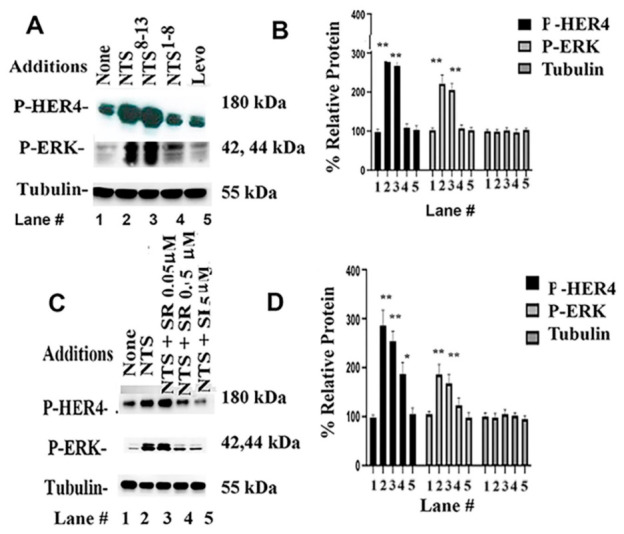
HER4 transactivation by NTS analogs. (**A**) The ability of NTS, NTS^8–13^, Levo, and NTS^1–8^ to alter P-HER4, P-ERK, and tubulin was determined. (**B**) The graph indicates the % relative protein for P-HER4, P-ERK, and tubulin in lanes 1–5. (**C**) The increase in P-HER4, P-ERK, and tubulin caused by NTS was inhibited by 0.5 or 5 μM but not 0.05 μM SR48692. (**D**) The graph indicates the % relative protein for P-HER4, P-ERK, and tubulin in lanes 1–5. There was no effect on tubulin. This experiment is representative of 2 others. The mean value + S.D. of 3 determinations is indicated; *p* < 0.01 ** and *p* < 0.05 * by ANOVA.

The ability of NTS ligands to alter HER4 tyrosine phosphorylation was investigated. Adding NTS or NTS^8–13^ but not NTS^1–8^ or Levo to NCI-H661 cells increased P-HER4 and P-ERK but had little effect on the tubulin control (Figure 3A). Adding NTS and NTS^8–13^ significantly increased P-HER4 to 286 and 259%, as well as P-ERK to 231 and 206%, respectively. SR48692, in a dose-dependent manner, inhibited the ability of NTS to increase P-HER4 and P-ERK (Figure 3C). SR48692 inhibited the increase in P-HER4 caused by adding NTS at 0.5 and 5 μM, but not at 0.05 μM dose. Figure 3D shows that adding NTS to NCI-H661 cells increased P-HER4 to 295% and P-ERK to 189%. See original images in Supplemental Figures S3. As a control, each sample had the same amount of tubulin. Similar results were obtained using NCI-H522 cells. The data indicate that NTS and NTS^8–13^ are NTSR1 agonists, but NTS^1–8^ or Levo are inactive. SR48692 antagonizes the increase in P-HER4 and P-ERK by NTS addition.

### 3.4. siRNA

**Figure 4:**
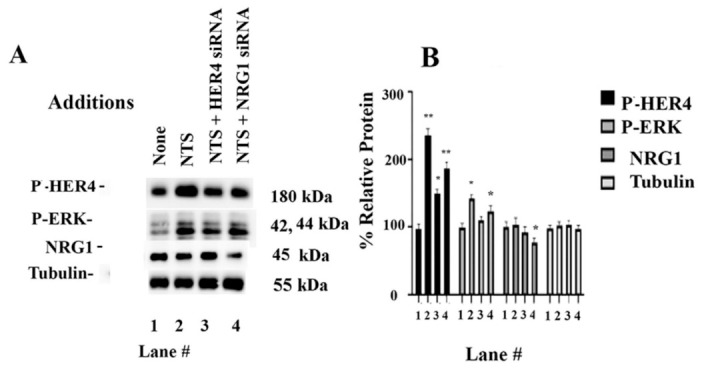
siRNA. Cells were treated with HER4 siRNA or NRG1 siRNA for 24 h. The cells were washed in SIT medium. (**A**) Adding 0.1 μM NTS for 10 min increased P-HER4, but the increase was significantly reduced when the cells were treated with HER4 siRNA or NRG1siRNA. P-ERK was increased by NTS, but the increase was reduced if the cells were treated with HER4 siRNA. NRG1 was decreased if the cells were treated with NRG1 siRNA but not NTS or HER4 siRNA. There was no effect on tubulin. (**B**) The graph indicates the % relative protein for P-HER4, P-ERK, NRG1, and tubulin in lanes 1–4. The mean value + S.D. of 3 determinations is indicated; *p* < 0.01 ** and *p* < 0.05 * by ANOVA.

SiRNA (10 nM) was added to NCI-H661 cells for 24 h. The treated cells were then placed in SIT medium. Adding (0.1 μM) NTS to NCI-H661 cells increased P-HER4, which was impaired if the cells were pretreated with HER4 siRNA or NRG1 siRNA (Figure 4A). Inactive siRNAs had no effect. Figure 4B shows that adding NTS to NCI-H661 cells increased P-HER4 significantly to 236%, which was reduced by NTSR1siRNA or NRG1siRNA treatment to 149% and 187%, respectively. HER4 siRNA inhibited P-ERK significantly. The addition of NRG siRNA significantly reduced the NRG1 to 69%. HER4 siRNA or NRG1 siRNA had little effect on the tubulin. control. See original images in Supplemental Figure S4. The results indicate that P-ERK and P-HER4 proteins, and NSCLC cells, are significantly reduced by HER4 siRNA, whereas NRG1 is reduced by NRG1 siRNA.

### 3.5. HER4 Phosphorylation Inhibitors

**Figure 5:**
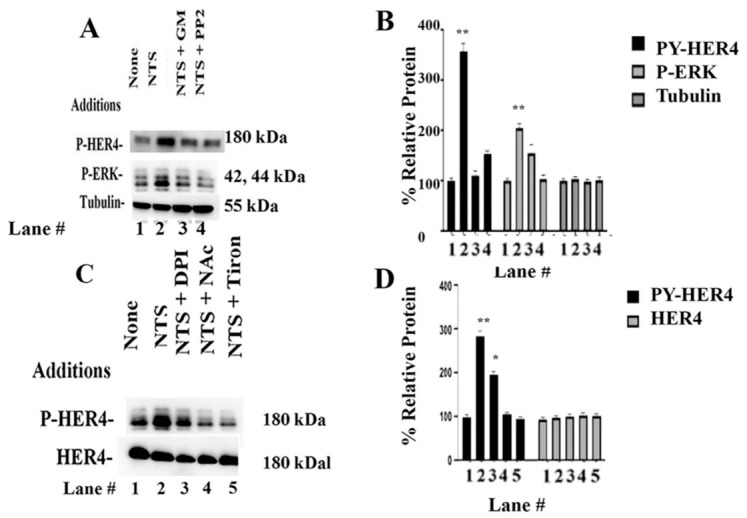
Transactivation inhibitors. (**A**) Adding NTS to NCI-H661 cells increases P-HER4 and P-ERK, which are inhibited by GM6001 or PP2. There was no effect on tubulin. (**B**) The graph indicates the % relative protein for P-HER4, P-ERK, NRG1, and tubulin in lanes 1–4. There was no effect on total HER4 (**B**). The graph indicates the % relative protein for P-HER4, P-ERK, and tubulin in lanes 1–4. (**C**) The ability of NTSR1 to regulate P-HER4 is impaired by DPI, NAc, or Tiron. There is no effect on HER4. (**D**) The graph indicates the % relative protein for P-HER4 and HER4 in lanes 1–5. The mean value + S.D. of 3 determinations is indicated; *p* < 0.01 ** and *p* < 0.05 * by ANOVA.

The ability of inhibitors to impair the NTSR1 transactivation of HER4 was investigated. Figure 5A shows that the Src inhibitor PP2 and the MMP inhibitor GM6001 impaired the ability of NTSR1 to regulate HER4 transactivation. Adding NTS to NCI-H661 cells increased P-HER4 to 357%, which was significantly decreased by PP2 and GM6001 to 110 and 153%, respectively. Adding NTS to NCI-H661 cells increased P-ERK levels to 204%, which were inhibited by PP2 or GM6001. PP2 or GM6001 had no effect on tubulin. Figure 5 shows that diphenylene iodonium (DPI is a NOX/DUOX inhibitor), N-acetylcysteine (NAc is an antioxidant), or Tiron (Tir is a superoxide scavenger) impaired the ability of NTSR1 to regulate HER4 transactivation. Figure 5D shows that NTS increased P-HER4 to 283%, which was significantly reduced to 196%. Pretreating NCI-H661 cells with DPI, NAc, or Tir had no effect on total HER4. See original images in Supplemental Figures S5 and S6. The results indicate that Src, MMP, and reactive oxygen species (ROS) are essential for NTS to increase HER4 phosphorylation.

### 3.6. Dimerization

**Figure 6:**
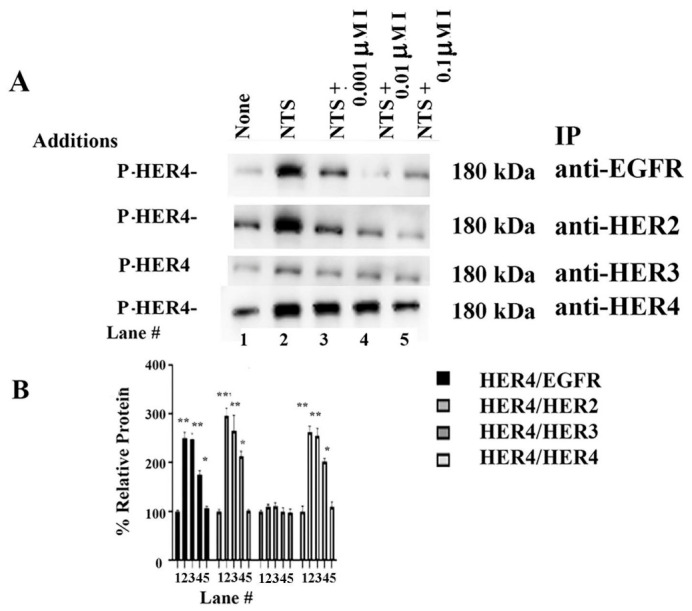
Dimerization. (**A**) NCI-H661 cells were incubated with 0.001, 0.01, or 0.1 μM ibrutinib (I) for 30 min, then treated with NTS for 10 min. The lysis extracts were immunoprecipitated with anti-EGFR, anti-HER2, anti-HER3, or anti-HER4 and analyzed by Western blot. The nitrocellulose was treated with P-HER4 antibody. (**B**) The graph shows the % relative protein for P-HER4 in lanes 1–5. This experiment is representative of 3 others. The mean value + S.D. of 3 determinations is indicated as *p* < 0.01, **; *p* < 0.05, * by ANOVA.

The presence of HER4 homodimers and heterodimers was investigated. NCI-H661 cells were treated with varying concentrations of Ibrutinib for 30 min. Then 0.1 μM NTS was added for 10 min. The samples were immunoprecipitated with anti-EGFR, anti-HER2, anti-HER3, or anti-HER4. The blots were then treated with P-HER4. NCI-H661 cells increased P-HER4 if the cells were immunoprecipitated with anti-EGFR, anti-HER2, and anti-HER4 but not anti-HER3 (Figure 6A). Figure 6B shows that adding NTS to NCI-H661 cells increased PY-HER4 homodimer levels to 278%. NTS increased the formation of HER4/EGFR and HER4/HER2 heterodimers to 297% and 284%, respectively. HER4 tyrosine kinase inhibitor, ibrutinib (I) at 0.001 μM, did not impair the ability of NTS to increase PY-HER4 (Figure 6B). Ibrutinib at 0.01 or 0.1 μM significantly inhibited the formation of HER4 homodimers as well as HER4/EGFR and HER4-HER2 heterodimers moderately and strongly, respectively. HER4/HER3 heterodimers were not detected. See original images in Supplemental Figure S7. The results indicate that NTS increases the formation of HER4 homodimers and HER4/EGFR as well as HER4/HER2 heterodimers.

### 3.7. Effect of PI3K and MEK Inhibitors

**Figure 7:**
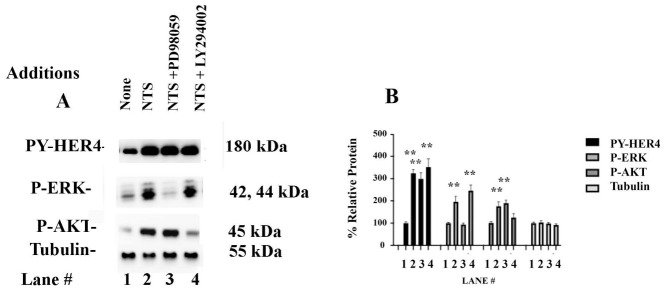
PI3K and MAPK. (**A**) NCI-H661 cells were incubated with 10 μM PD 98059 or LY294002 for 30 min. After adding 0.1 μM NTS for 10 min. P-HER4, P-ERK, P-AKT, and Tubulin were determined. (**B**) The graph indicates the % relative protein for P-HER4, P-ERK, P-AKT, and tubulin in lanes 1–4. The mean value + S.D. of 3 determinations is indicated; *p* < 0.01 ** by ANOVA.

P-PI3K and P-AKT are associated with increased cancer cell survival; P-MEK and P-ERK are associated with increased cancer cell proliferation [18]. Pretreatment of NCI-H661 cells with the PI3K inhibitor LY294002 impaired the phosphorylation of the ability of NTS to phosphorylate AKT but did not affect P-HER4, P-ERK, or tubulin (Figure 7A). Pretreatment of the NCI-H661 cells with the MEK inhibitor PD98059 reduced the ability of NTS to increase PY-ERK but had no effect on PY-HER4, P-AKT, or tubulin. Adding NTS to NSCLC cells increased P-AKT to 161%, which was inhibited significantly by 10 μM LY295020 (Figure 7B). Adding NTS to NCI-H661 cells increased P-ERK to 208%, which was significantly inhibited by 10 μM PD98059. Adding NTS increased NSCLC cells, and PY-HER4 to 312%, which was not affected by PD98059 or LY294002. See original images for Supplementary Figure S8. The results indicate that PI3K and MEK inhibitors do not impair NTSR1’s ability to regulate HER4 transactivation.

### 3.8. Proliferation

Proliferation of NSCLC cells was investigated using the MTT and clonogenic assay. Table 1 shows that adding NTS or NRG1 significantly increased the NCI-H661 colony number from 100% to 141% and 151%, respectively. Similarly, adding NTS or NRG1 to NCI-H522 cells significantly increased the number of colonies to 133% and 155%, respectively. Adding siRNA for HER4 or NRG1 significantly reduced NCI-H661 colony number from 100% to 66 and 79%, respectively. Adding siRNA for HER4 or NRG1 decreased NCI-H522 colony number to 71% and 82%, respectively. Adding SR48692 or ibrutinib significantly decreased the NCI-H661 colony number from 100% to 59% and 62%, respectively. Adding SR48692 or ibrutinib decreased NCI-H522 colony number from 100% to 71 and 82%, respectively. Adding SR48692 and ibrutinib together significantly reduced the NCI-H661 colony number from 100% to 52%. Adding SR48692 and ibrutinib significantly reduced the NCI-H522 colony number from 100% to 52%. The results indicate that NTS or NRG1 increase NSCLC colony formation, whereas SR48692 and ibrutinib reduce colony formation.

**Figure 8:**
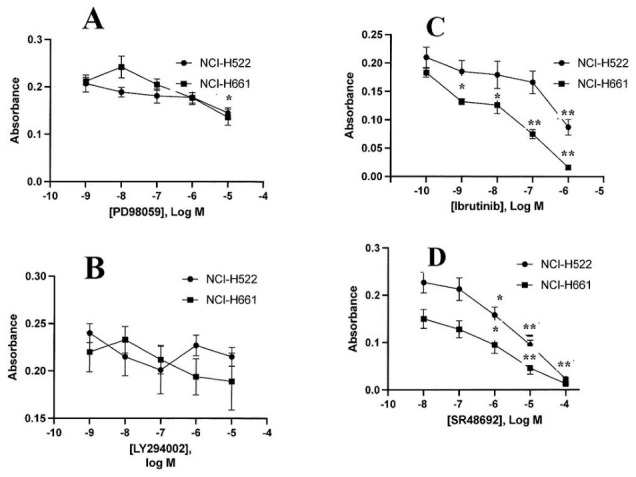
MTT assay. The ability of varying doses of (**A**) PD59095, LY294002 (**B**), Ibrutinib (**C**), or SR48692 (**D**) to inhibit the proliferation of NCI-H522 and NCI-H661 cells is indicated. The mean value + S.D. of 8 determinations is indicated; *p* < 0.01 ** and *p* < 0.05 * by ANOVA.

The ability of inhibitors to alter the growth of NSCLC cells was investigated using the MTT assay. Figure 8A shows that 10 μM PD98059, but not lower doses, inhibited the proliferation of NCI-H522 and NCI-H661 cells. In contrast, LY294002, which inhibits PI3K, had no effect on NSCLC growth at any concentration tested (Figure 8B). Adding ibrutinib, a TKI inhibitor, to NCI-H661 inhibits growth at concentrations of 0.001, 0.01, 0.1, and 1 μM. In contrast, ibrutinib significantly inhibited NCI-H522 cells at a 1 μM dose but not at lower concentrations. NSCLC cell lines such as NCI-H522, which have high WNT5A expression, impair the effects of ibrutinib [22]. Adding SR48692 to NSCLC cells inhibited the proliferation of NCI-H522 and NCI-H661 cells significantly at 1, 10, and 100 μM concentrations, but not at lower doses. The IC_50_ of SR48692 for NCI-H522 and NCI-H661 cells was 2.8 and 5.2 μM. The results indicate that SR48692 inhibits the proliferation of NCI-H522 and NCI-H661 NSCLC cells with similar affinity, whereas ibrutinib is a more potent inhibitor of NCI-H661 cells than of NCI-H522 cells.

## 4. Discussion

HER4, which contains 1308 amino acids, has a single TM domain and is activated by numerous ligands, including NRG1, NRG2, NRG3, NRG4, heparin-binding epidermal growth factor, and betacellulin [32]. NRG1 is a 44 kDal glycoprotein which is stored in the plasma membrane and released from lung cancer cells [33]. After binding NRG-1, HER4 can be phosphorylated at Y^1284^ or Y^1056^, activating ERK and PI3K, respectively. NRG-1 activation of HER4 leads to the formation of HER4 homodimers or HER4-HER2 heterodimers [18]. Figure 1 shows that HER4 and NTSR1 proteins are present in all six NSCLC cell lines examined. NTS and NTSR1 mRNA were detected in approximately 67% of the NSCLC patient biopsy specimens examined [17]. A surprising finding is that NTSR1 and NTSR3 are co-expressed in colon cancer cells. Through immunocytochemistry, NTS, NTSR1, and NTSR3 are expressed in colorectal tumors but not in normal tissue [34]. NTSR3 augments cell adhesion and migration in neuroendocrine tumors by upregulating Src and by phosphorylating focal adhesion kinase (FAK) [35]. It remains to be determined if NTSR3 functions as a co-receptor for NTSR1 in lung cancer [36].

Table 2 shows the expression of numerous genes in NSCLC cells. The EGFR, HER2, HER4, and NTSR1 are expressed in all 6 cell lines examined. HER3 is present in NCI-H322, A549, H1299, and NCI-H2073 cells but not NCI-H522 or NCI-H661 cells. Mutated K-RAS is present in A549 cells. Mutated EGFR is present in NCI-H2073 cells. It remains to be determined if the mutated KRAS of the mutated EGFR has any effect on the NTSR1 regulation of HER4.

NTS and NTS^8–13^ but not Levo or NTS^1–8^ bind with high affinity to NTSR1, resulting in HER4 transactivation (Figure 3). NTSR1 interacts with Gq11, causing PI metabolism, and the released DAG activates PKC, whereas the IP_3_ released increases cytosolic Ca^2+^. Figure 2 shows that NTS or NT^8–13^, but not Levo or NTS^1–8^ increases cytosolic Ca^2+^ in NSCLC cell line NCI-H661. SBI-553 is an NTSR1 biased agonist [9]. Adding SBI-553 to cells increases the phosphorylation of the NTSR1 by the G protein-coupled receptor kinase 2 (GRK) [39]. SBI-553 changes the conformation of NTSR1, increasing interaction with GRK2 [40]. β-arrestin interacts with the phosphorylated NTSR1, leading to the internalization of NTSR1, minimizing interaction with Gq [9].

NTSR1 regulates P-HER4 transiently in a ROS-dependent manner. Adding NTS to NSCLC cells increases ROS to 228%, whereas H_2_O_2_ increases ROS to 1154% [28]. DPI, NAc, or Tiron significantly inhibited the ability of NTS to increase HER4 tyrosine phosphorylation (Figure 5). DPI inhibits NADPH oxidase (NOX) and dual oxidase (Duox) enzymes, reducing production of ROS [41]. When essential Cys amino acids in protein tyrosine phosphatase are oxidized, RTK phosphorylation increases [42]. Src inhibitors as well as MMP inhibitors impair the ability of NTSR1 to transactivate HER4 (Figure 5). Adding NTS to NSCLC cells increases SRC phosphorylation, which is inhibited by U73122, a PLC inhibitor [16]. Activated Src phosphorylates FAK, increasing cancer cellular migration. Adding NTS to NSCLC cells increases P-FAK [43]. It remains to be determined if NTS impairs protein tyrosine phosphatase activity.

NRG-1 was detected in 9 of 9 NSCLC cell lines tested [21]. The 222 amino acid NRG-1 is released from the plasma membrane after metabolism of the NRG1 precursor by MMP [44]. NTS increases MMP9 activity, resulting in increased gastric cancer cellular migration [45]. NRG1 can activate HER3 and HER4. Because NCI-H522 or NCI-H661 cells have little HER3, the NRG1 activates HER4 primarily in NCI-H522 and NCI-H661 cells. NRG-1 fusions occur in some NSCLC patients, leading to increased RTK activity [46,47] and decreased patient survival.

HER4 can have genetic mutations or splice variants (SV). Splice variants of the HER4 extracellular juxtamembrane domain result in JMa and JMb, whereas SVs in the cytosolic domain result in CYT1 and CYT2 [48]. mRNA for JMa is abundant in NSCLC cells [21], and it is metabolized by tumor necrosis factor and gamma-secretase into an intracellular cytosolic domain, which affects gene transcription. HER4 is mutated in both the extracellular juxtamembrane domain and the tyrosine kinase domain, resulting in increased proliferation. The extracellular domain of HER4 includes domains I and III, which are important for ligand binding. Domains II and IV are enriched in cysteine amino acids and are important in dimerization. When NRG1 binds to HER4, the HER4 conformation changes from a tethered to an extended state [18]. After the conformational change, domains II and IV are close to one another, leading to the dimerization of HER4. Figure 6 shows that after the addition of NTS, HER4/HER4 homodimers, HER4/EGFR, and HER4/HER2 heterodimers form. HER4 mutations occur in the extracellular ligand-binding domain of HER4, increasing HER4/HER2 heterodimer formation [48].

SR48692 is an NTSR1 antagonist that inhibits HER tyrosine phosphorylation caused by adding NTS to NSCLC cells (Figure 3). The growth of A549 xenografts in nude mice is impaired by NTSR1 siRNA [30]. HER4 siRNA reduced the amount of P-HER4 (Figure 4). NTSR1 siRNA reduced EGFR tyrosine phosphorylation and NTSR1 levels [16]. SR48692 is synergistic with gefitinib (EGFR TKI) in inhibiting NSCLC cell growth [17]. SR48692 improves the response to carboplatin in patients with ovarian cancer [49]. SR48692 increases the response to lapatinib (TKI) or metformin in breast cancer patients [50]. Reduced NTSR1 levels increase progression-free survival and overall survival of NSCLC patients [17]. NTS and NRG1 increase NSCLC colony formation, whereas SR48692 and ibrutinib inhibit proliferation (Table 1). Ibrutinib is used to treat patients with chronic lymphocyte leukemia [51] but inhibits NSCLC proliferation [22]. It remains to be determined if ibrutinib is synergistic with SR48692 in inhibiting the growth of NSCLC tumors.

Current efforts focus on determining if NTSR1 is a biomarker for cancer [52]. Using PET imaging techniques, ^68^Ga-DOTA-NT-20.3 is used to differentiate human pancreatic ductal pancreatic adenocarcinoma from pancreatitis [53]. A ^177^Lu-NTS analog is being tested using animal models of colorectal and pancreatic cancer [54]. It remains to be determined whether NTS analogs serve as theragnostic agents for the treatment of lung cancer patients.

## 5. Conclusions

NTSR1 mediates HER4 transactivation in lung cancer cells. The increase in P-HER4 after the addition of NTS is impaired by ibrutinib and SR48692. NTS or NRG1 stimulate the clonal growth of NSCLC cells, whereas SR48692 or ibrutinib inhibit growth in vitro. It remains to be determined if SR48692, when combined with ibrutinib, will strongly inhibit the growth of NSCLC in vivo.

## Figures and Tables

**Table 1:** Clonogenic assay.

Cell Line Addition	% NCI-H661 Basal	% NCI-H522 Basal
None	100 ± 12	100 ± 9
HER4 siRNA	67 ± 16 *	66 ± 20 *
NRG-1 siRNA	73 ± 14 *	79 ± 8
SR48692	59 ± 9 **	71 ± 9 *
Ibrutinib	62 ± 12 *	82 ± 12
SR + I	52 ± 12 **	52 ± 9 **
NRG-1	141 ± 24 *	133 ± 9 *
NTS	151 ± 15 **	155 ± 18 *

The mean value ± S.D. of 3 determinations was calculated; *p* < 0.05, *; *p* < 0.01, ** by ANOVA relative to no additions.

**Table 2:** Gene expression in NSCLC cell lines.

NSCLC Cell Line	EGFR	HER2	HER3	HER4	NTSR1	KRAS Mutations	EGFR Mutations
NCI-H322	+	+	+	+	+	-	-
NCI-H522	+	+	-	+	+	-	-
A549	+	+	+	+	+	+	-
NCI-H661	+	+	-	+	+	-	-
NCI-H1299	+	+	-	+	+	-	-
NCI-H2073	+	+	+	+	+	-	+

Expression of oncogenes in 6 NSCLC cell lines by RT-PCR [37,38]; positive expression, +; negative expression, -.

## Data Availability

The raw data supporting the conclusion of this article will be made available by the authors without reservation.

## Supplementary Materials

The following supporting information can be downloaded at: https://www.mdpi.com/article/10.3390/biology15090686/s1.
