# Supplementary figures and images for: Neurotensin Receptor 1 Regulates HER4 Tyrosine Phosphorylation in Lung Cancer Cells
Source: Biology (Basel). 2026 Apr 28;15(9):686. doi: 10.3390/biology15090686 (PMC13162706; doi:10.3390/biology15090686)

**Fig. S7**

**A1**

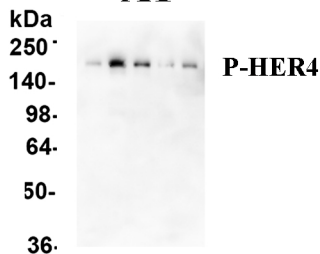

**A2**

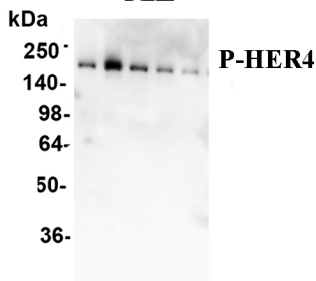

**A3**

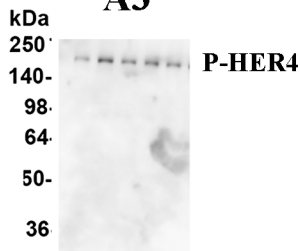

**A4**

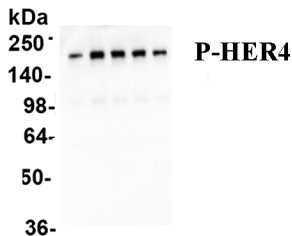

Supplement: Supplementary file 1 [file biology-15-00686-s001.zip › biolmoodyFigS7.pdf]

**Fig. S1**

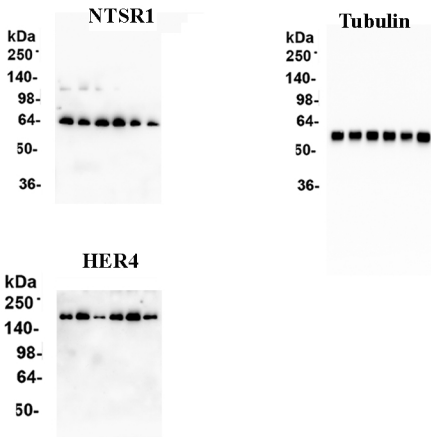

Supplement: Supplementary file 1 [file biology-15-00686-s001.zip › biolmoodytFigS1.pdf]

**Fig. S2**

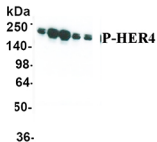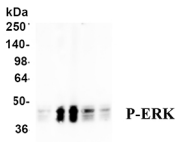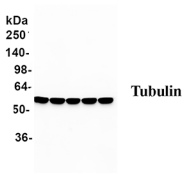

Supplement: Supplementary file 1 [file biology-15-00686-s001.zip › biolmoodytFigS2F.pdf]

**Fig. S3**

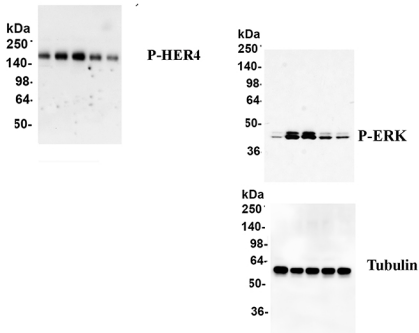

Supplement: Supplementary file 1 [file biology-15-00686-s001.zip › biolmoodytFigS3.pdf]

**Fig. S4**

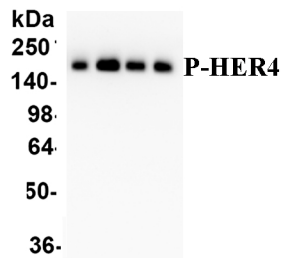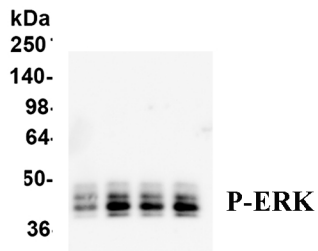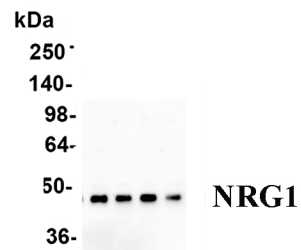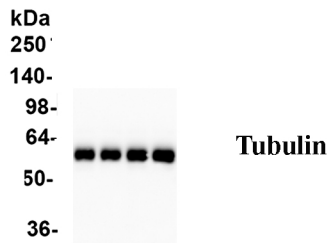

Supplement: Supplementary file 1 [file biology-15-00686-s001.zip › biolmoodytFigs4.pdf]

**Fig. S5**

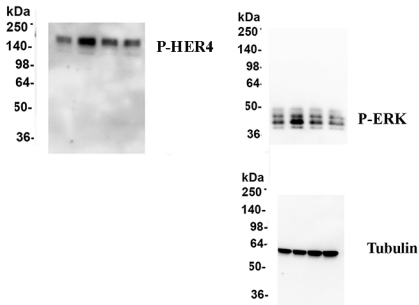

Supplement: Supplementary file 1 [file biology-15-00686-s001.zip › biolmoodytFigS5.pdf]

**Fig. S6**

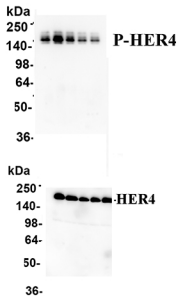

Supplement: Supplementary file 1 [file biology-15-00686-s001.zip › biolmoodytFigS6.pdf]

**Fig. S8**

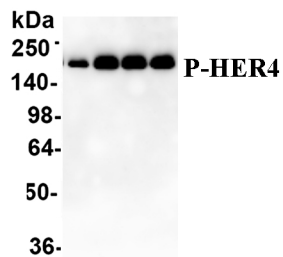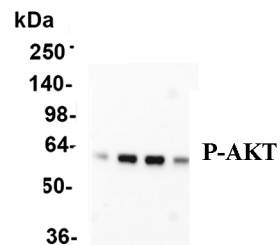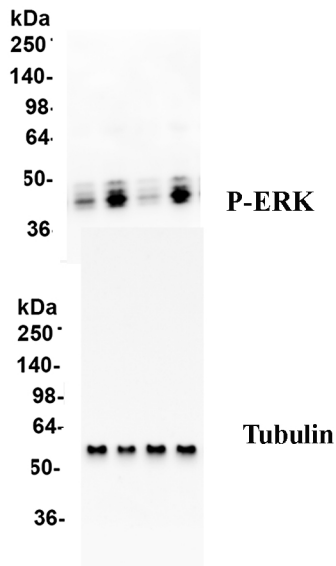

Supplement: Supplementary file 1 [file biology-15-00686-s001.zip › biolmoodytFigS8.pdf]
